# Supplementary material for: Coupling of growth rate and developmental tempo reduces body size heterogeneity in C. elegans
Source: Nat Commun. 2022 Jun 6;13:3132. doi: 10.1038/s41467-022-29720-8 (PMC9170734; doi:10.1038/s41467-022-29720-8)
Supplement: Supplementary file 1 — Supplementary Information [file 41467_2022_29720_MOESM1_ESM.pdf]

Supplementary Information

Coupling of growth rate and developmental tempo reduces body size heterogeneity in *C. elegans*

Klement Stojanovski[1], Helge Großhans[*,2,3], Benjamin D. Towbin[*,1,2]

[1] University of Bern, Bern, Switzerland

[2] Friedrich Miescher Institute for Biomedical Research (FMI), Basel, Switzerland

[3] University of Basel, Basel, Switzerland

* correspondence to: benjamin.towbin@unibe.ch, helge.grosshans@fmi.ch

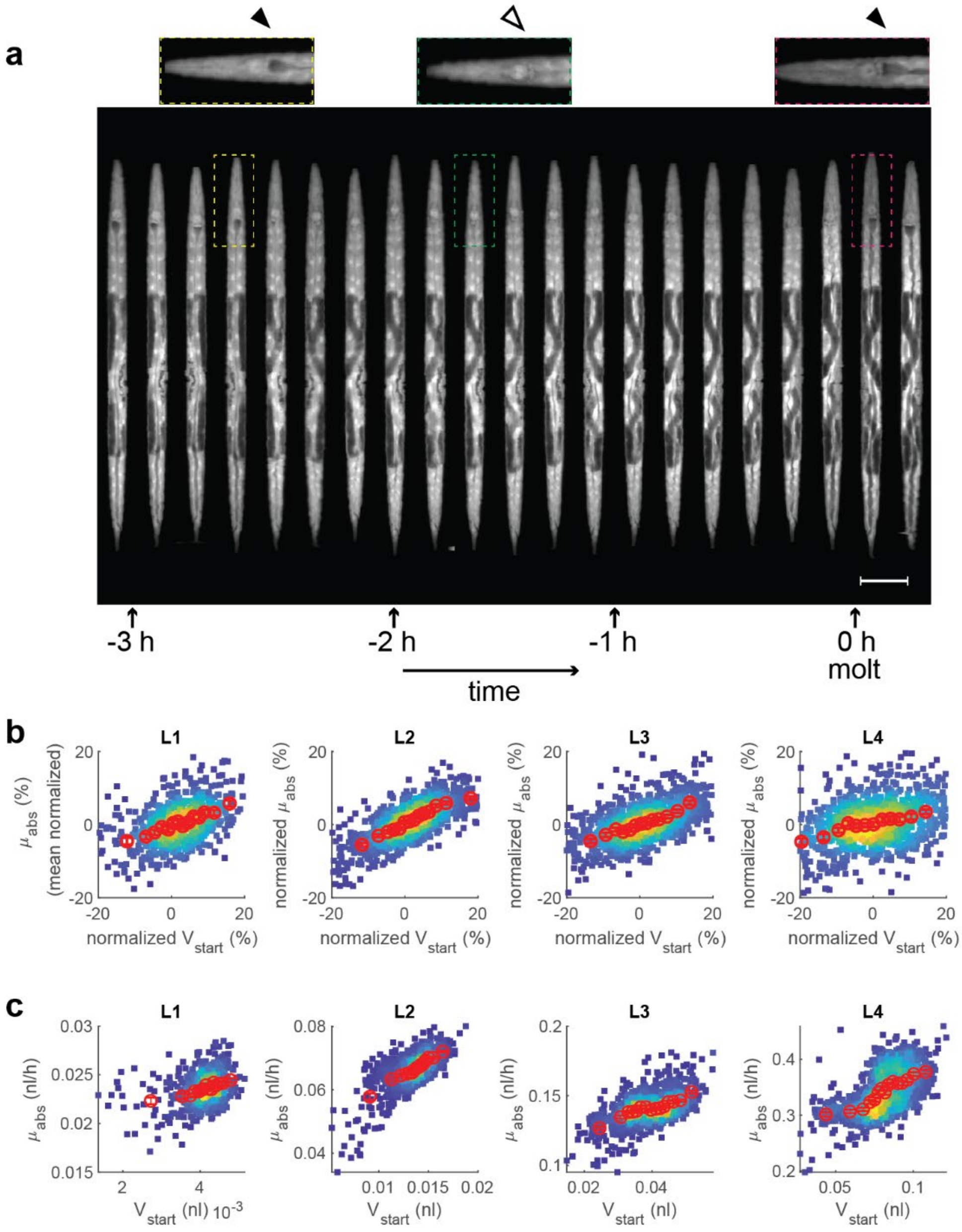

Supplementary Figure 1. Quantification of volume growth of *C. elegans*

(related to Figure 1)

a. GFP signal of a representative time series of straightened images of an individual starting 3 hours before the M4 moult. Yellow box: last time point before lethargus. Green box: time point during lethargus. Pink box: First time point after lethargus. Arrowheads highlight an opening of the intestine posterior to the pharynx (dark area in yellow and pink box) that is not visible during lethargus (green box) when the intestine is constricted and feeding stops. Dark areas in the center of the worm correspond to the gonadal arms, where the *eft-3::gfp* transgene is silenced. Pharyngeal constriction was observed in 10/10 animals inspected. Scale bar: 0.1mm

b. Scatter plot of absolute growth rate vs. the volume at larval stage entry shown as deviation from population mean. Red circles are the moving average along the x-axis.

c. As (b), but with unnormalized volumes and growth rates.

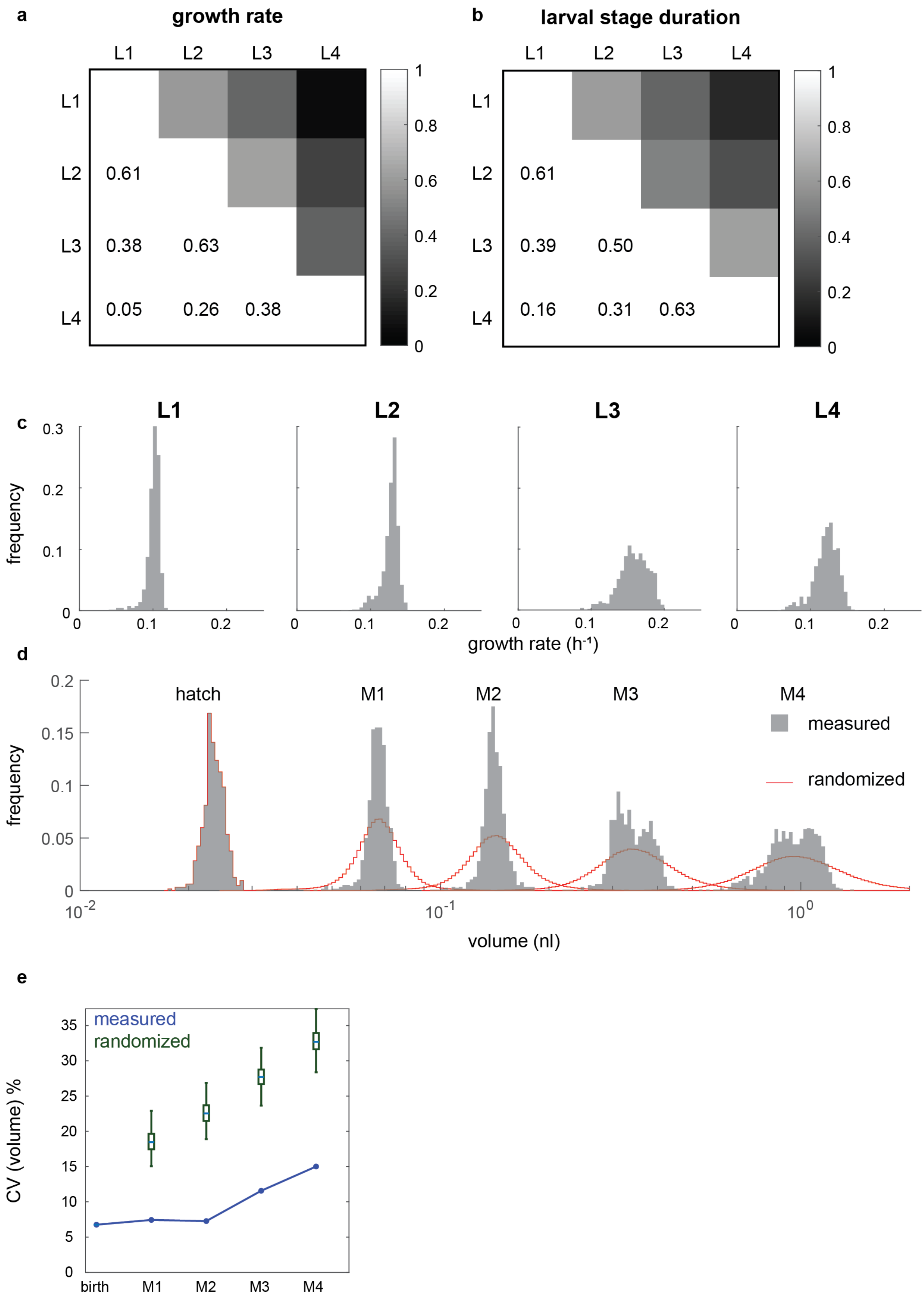

Supplementary Figure 2. Measurement of body volume divergence without normalization

(related to Figure 2)

a, b. Pearson correlation coefficients of growth rates (a) and larval stage durations (b) between larval stages.

c. Histogram of growth rate distribution without normalization to mean of day-to-day repeat.

d. Histogram of volume distribution without normalization to mean of day-to-day repeat. Red line indicates volume divergence expected by random shuffling of growth rates and duration of larval stages.

e. CV of volume for the four larval moults without normalization to mean of day-to-day repeat. Blue line shows measured CV. Green: distribution of randomly shuffled controls for 1000 iterations of random shuffling. Boxplots: central line: median, box: interquartile ranges (IQR), whisker: ranges except extreme outliers (>1.5*IQR).

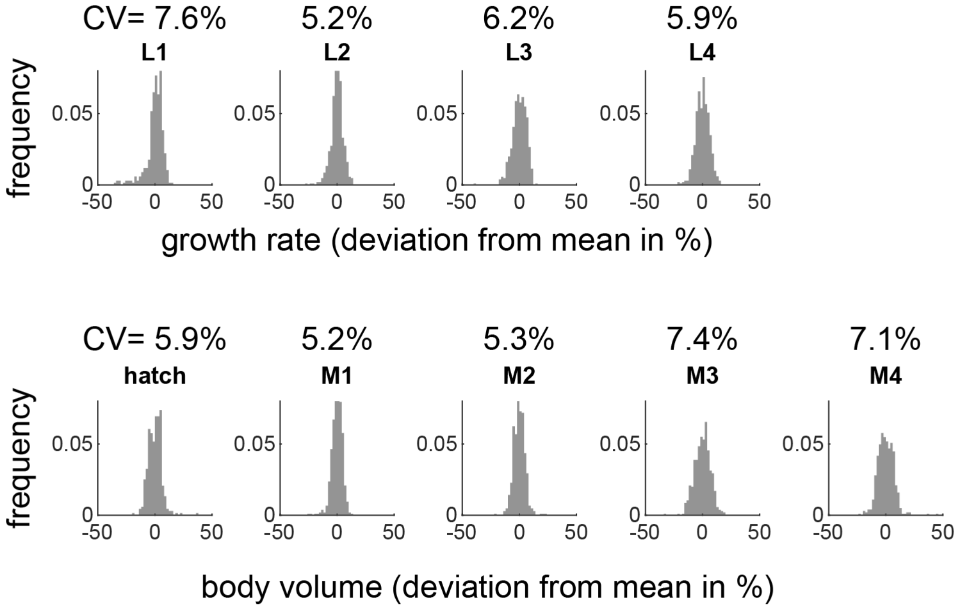

Supplementary Figure 3. Volume divergence among individuals and growth rate heterogeneity at 20°C

(related to Supplemental Figures 2)

(top) Histogram of the growth rate deviations in % from the population mean measured at indicated larval stages at 20°C. The growth rate of each individual and larval stage was determined by a linear regression to log(volume) against time excluding the first 10% and the last 25% of the larval stage.

(bottom) Histogram of body volume deviations in % from the mean measured at birth and indicated larval moults at 20°C.

Coefficients of variation are indicated above each panel.

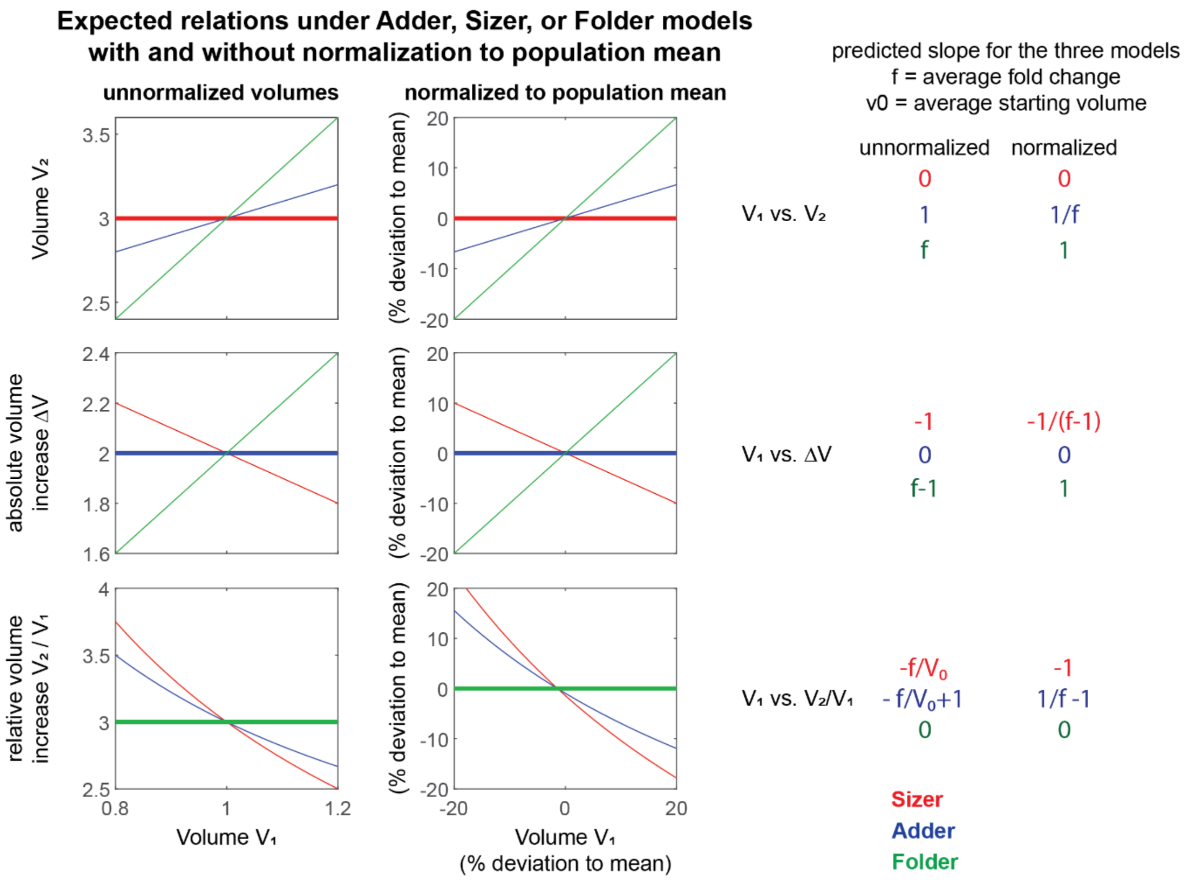

Supplementary Figure 4. Effect of normalization to population mean on sizer, adder, folder relations

(related to Figure 3)

Panels show the expected relations between V_1 and V_2 (top), V_1 and ΔV (middle), V_1 and FC_V (volume fold change) for sizers, adders (top), and folder without normalization not the population mean (left) and plotted as % deviation from the population mean (right). Table on the right shows theoretically predicted slopes. f is the average volume fold change per larval stage. V_0 is the average starting volume. For cases with non linear relation ship (bottom panels), the value for the slope at the population average is shown. red shows sizer, blue: adder, green: folder.

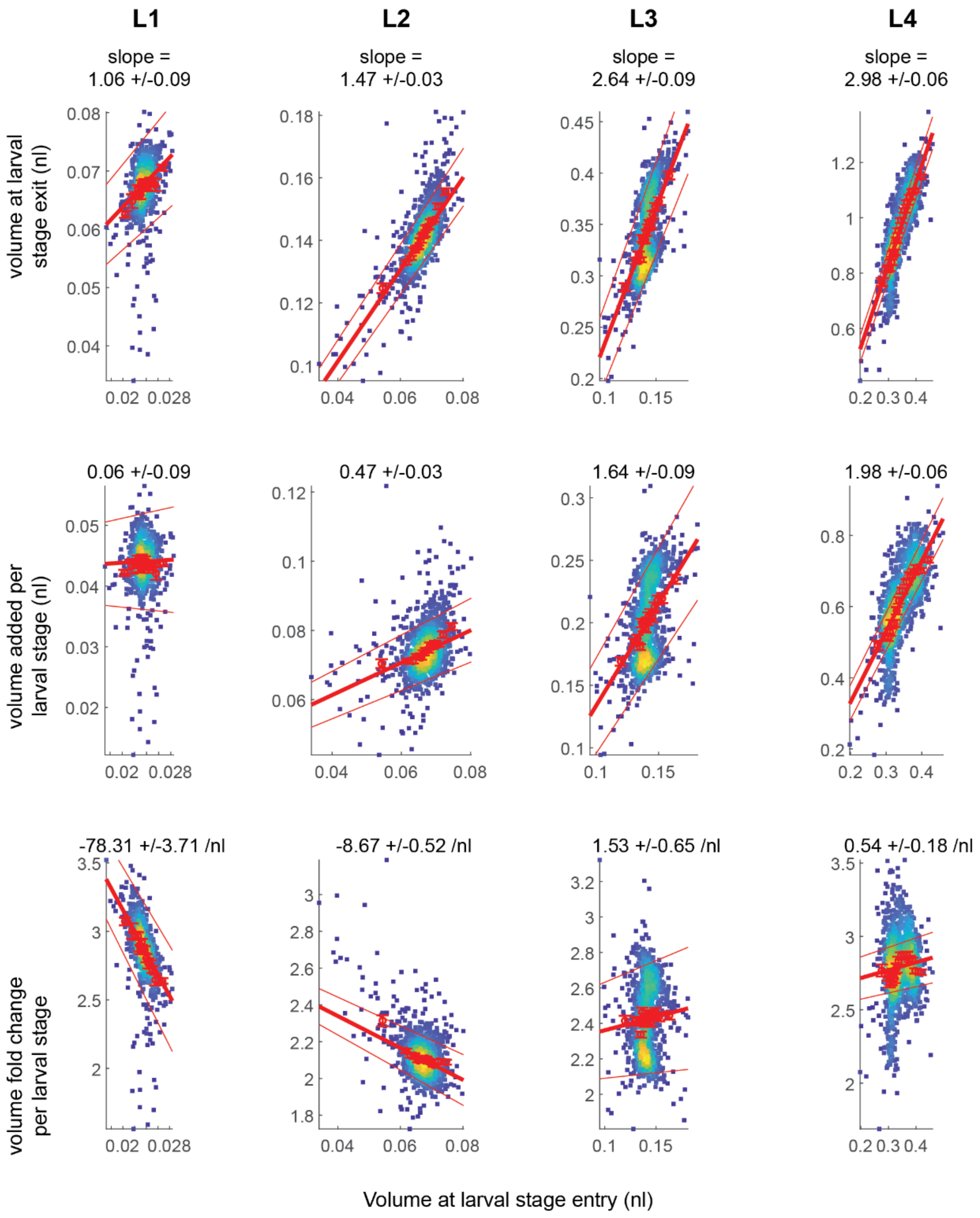

Supplementary Figure 5. adder, sizer, folder models for unnormalized data

(related to Figure 4)

Scatter plot of the volumes at larval stage entry and larval stage exit (top), the absolute volumes added per larval stage (middle), and volume fold changes (bottom). Data was not normalized to mean of day-to-day repeat. Red circles: moving average along the x-axis. Thick red trendline: robust linear regression. Thin red lines are 95% confidence interval of the fit. Slope +/- 95% CI of trendline are indicated above each panel.

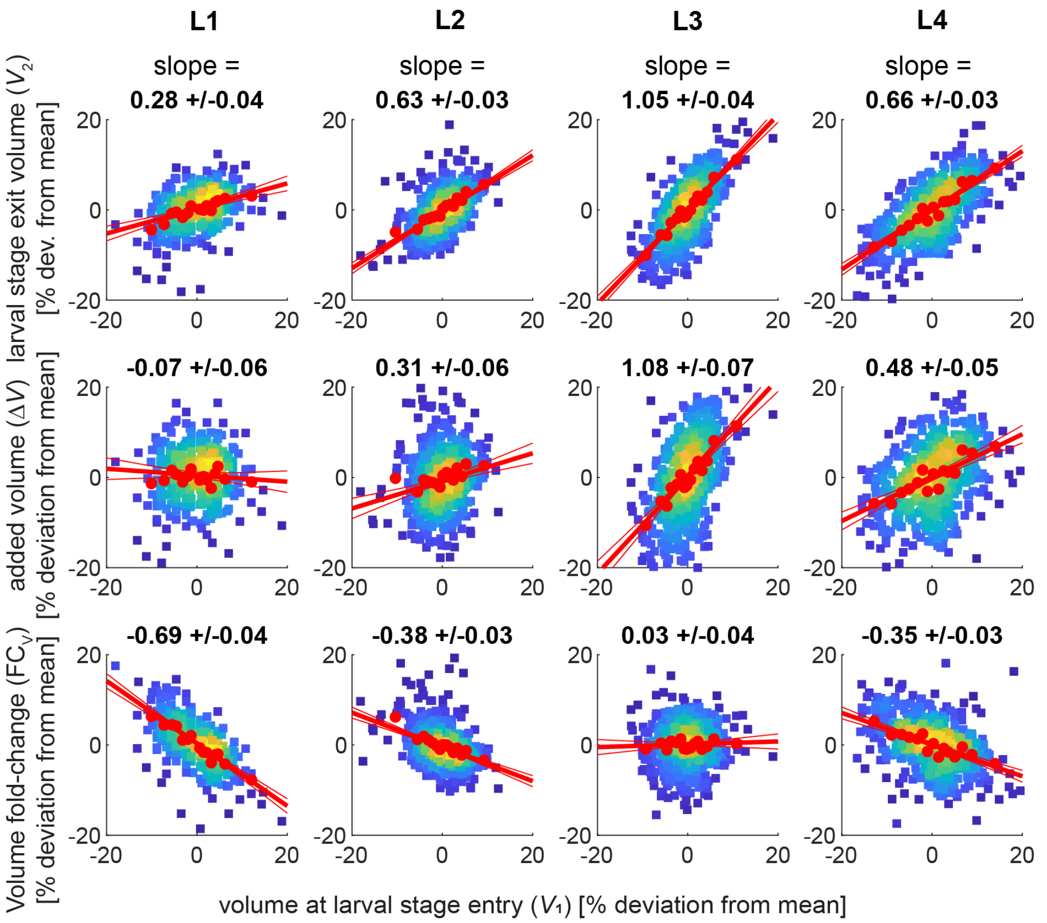

Supplementary Figure 6. adder, sizer, folder models at 20 °C

(related to Figure 4)

Scatter plot of volume at larval stage entry vs. volume at larval stage exit shown as % deviation from the mean for indicated larval stages. Colour indicates point density. Red circles are a moving average along x-axis. Thick red trendline: robust linear regression to the data (see methods). Thin red lines are 95% confidence interval of the fit. Slope +/- 95% CI of trendline are indicated above each panel.

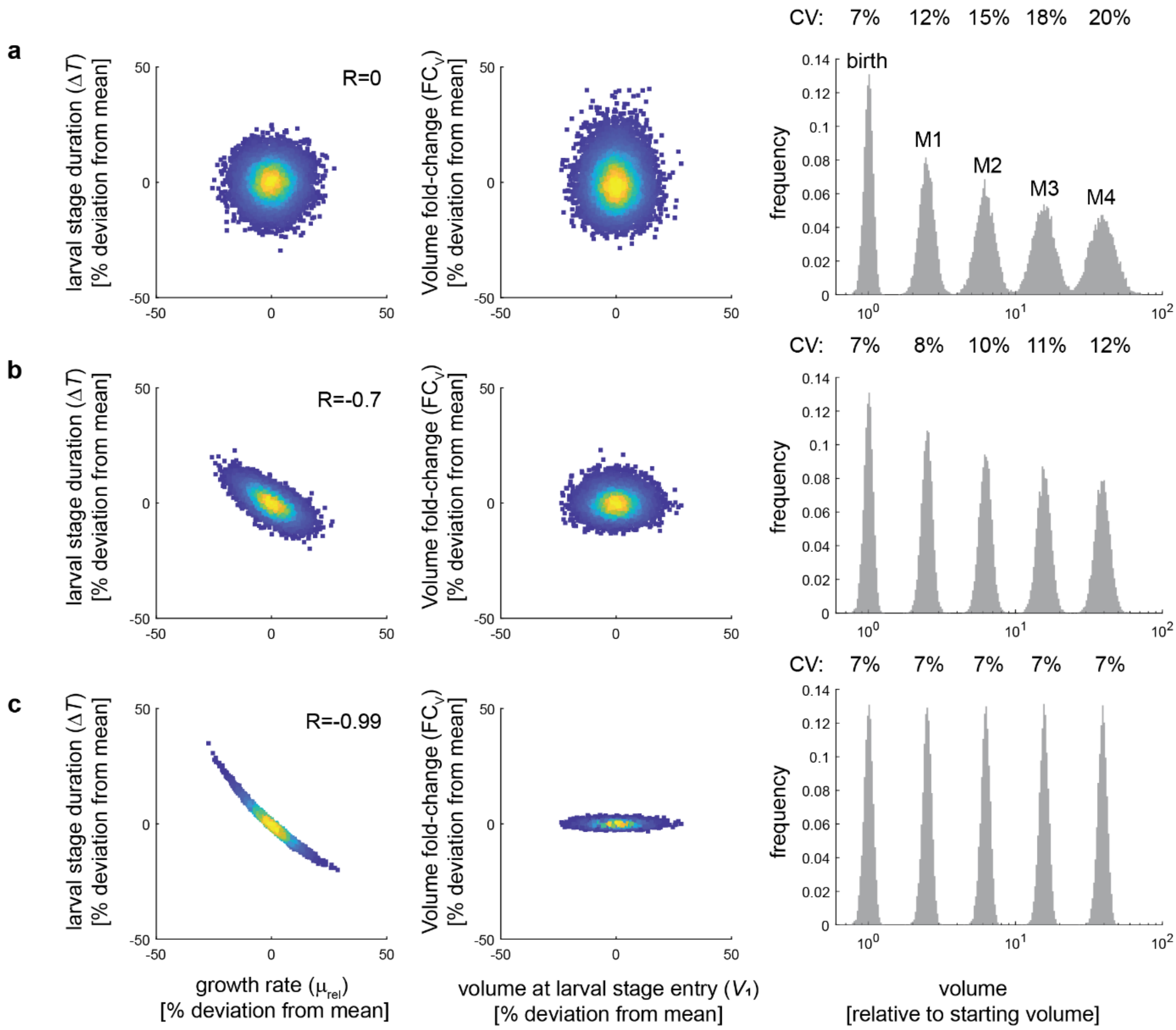

Supplementary Figure 7. Simulation of volume divergence as a function of anti-correlation between growth rate and duration of development

(related to Figure 4)

10,000 individuals were randomly assigned a starting volume (V_1), a growth rate (μ), and a larval stage duration (ΔT) drawn from normal distributions with CV=7% and $<\mu>$ = 0.11/h, $<\Delta T>$ = 8.32h. Volume fold change was computed from these parameters and the process was re-iterated 4 times. Correlation coefficient between μ and ΔT: (a) R = 0, (b) R= -0.7, or (c) R = -0.99. The folder occurs independently of the correlation between μ and ΔT (middle panel) but volume divergence is reduced when μ and ΔT are anti-correlated (right panel).

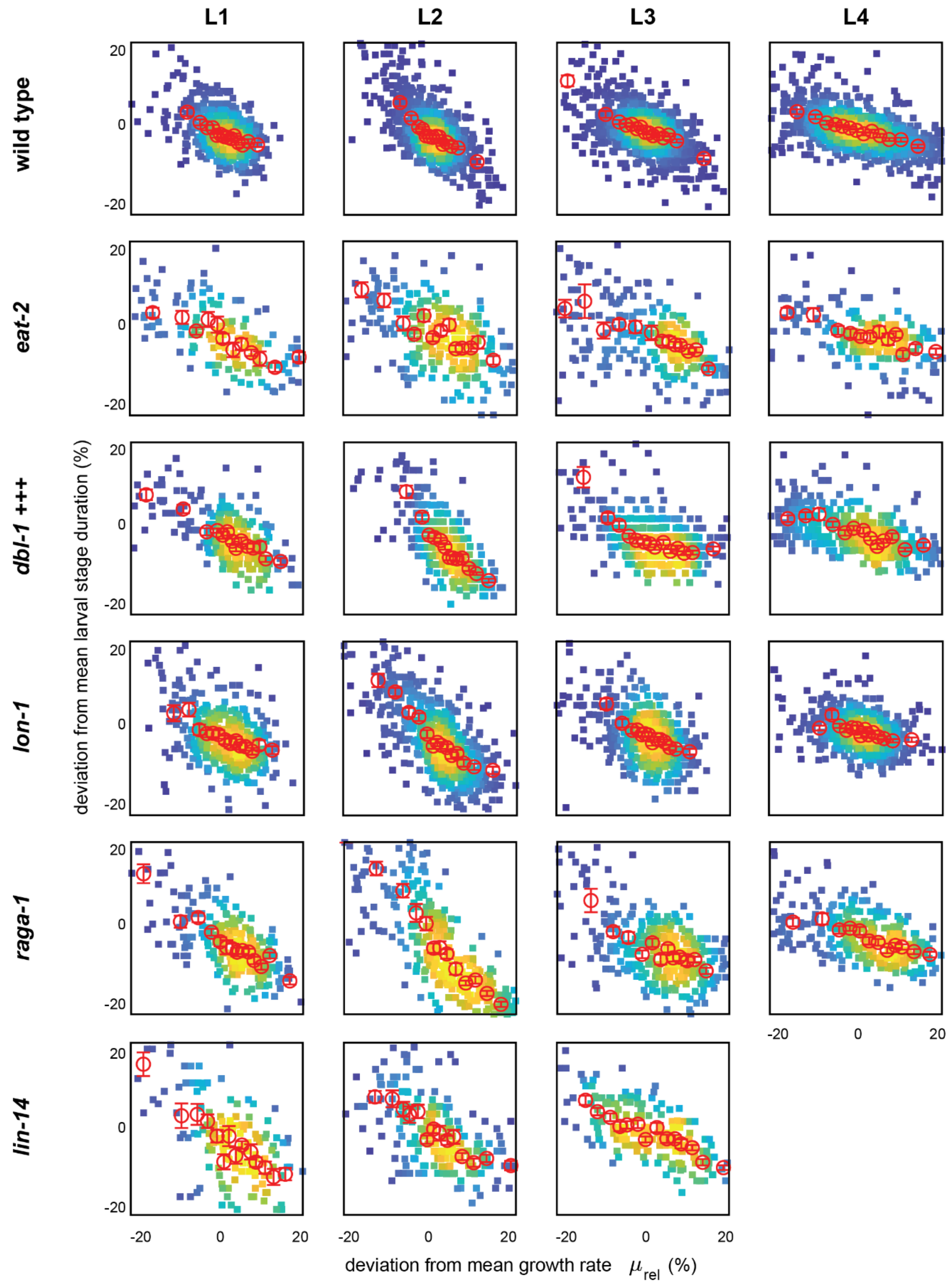

Supplementary Figure 8. Growth rate and larval stage duration remain anti-correlated in mutant strains

(related to Figure 5)

Scatter plot of growth rate vs. larval stage duration shown as % deviation from the mean for indicated mutants. Colour indicates point density. Red circles are moving average along x-axis +/- SEM. Number of animals (n) for L1 to L4 from number of independent experiments (m): wild type: n = 639, 1142, 1144, 1095; m = 10. *eat-2*: n = 270, 439, 440, 417; m = 3. *dbl++*: n = 433, 643, 650, 640; m = 2. *lon-1*: n = 450, 712, 716, 701; m = 7. *raga-1*: n = 347, 504, 501, 483; m = 3. *lin-14*: n = 288, 410, 387, n/a. m = 2

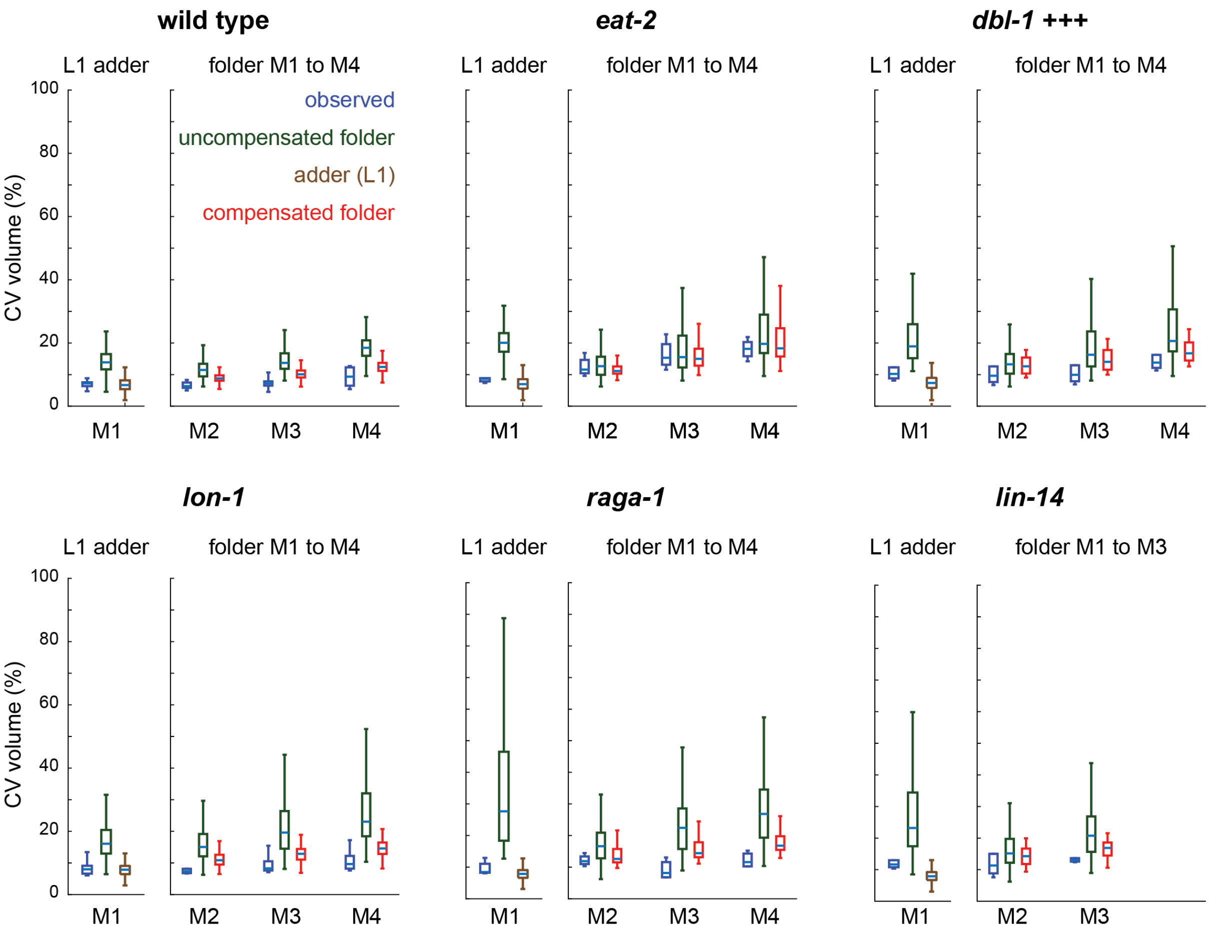

Supplementary Figure 9. Mutants with perturbed growth rate do not diverge more than expected in volume

(related to Figure 5)

Comparison of CV of volume to adder and folder models for indicated mutant backgrounds. Blue: box plot of the average CV measured experimentally in the different day-to-day repeats. Green: expected CV based on 1000x random shuffling of growth rate and larval stage duration. Brown: CV expected from adder model during L1. Red: CV expected from compensated folder model during L2 to L4. Randomizations were done separately for L1 and for L2 to L4 starting from measured volume distributions at birth and at M1, respectively to test separately for the impact of the folder compensation and the L1 adder on volume divergence. n = 10 (wild type), 3 (*eat-2*), 2 (*dbl-1++*), 7 (*lon-1*), 3 (*raga-1*), 2 (*lin-14*) independent experiments, Boxplots: central line: median, box: interquartile ranges (IQR), whisker: ranges except extreme outliers (>1.5*IQR).

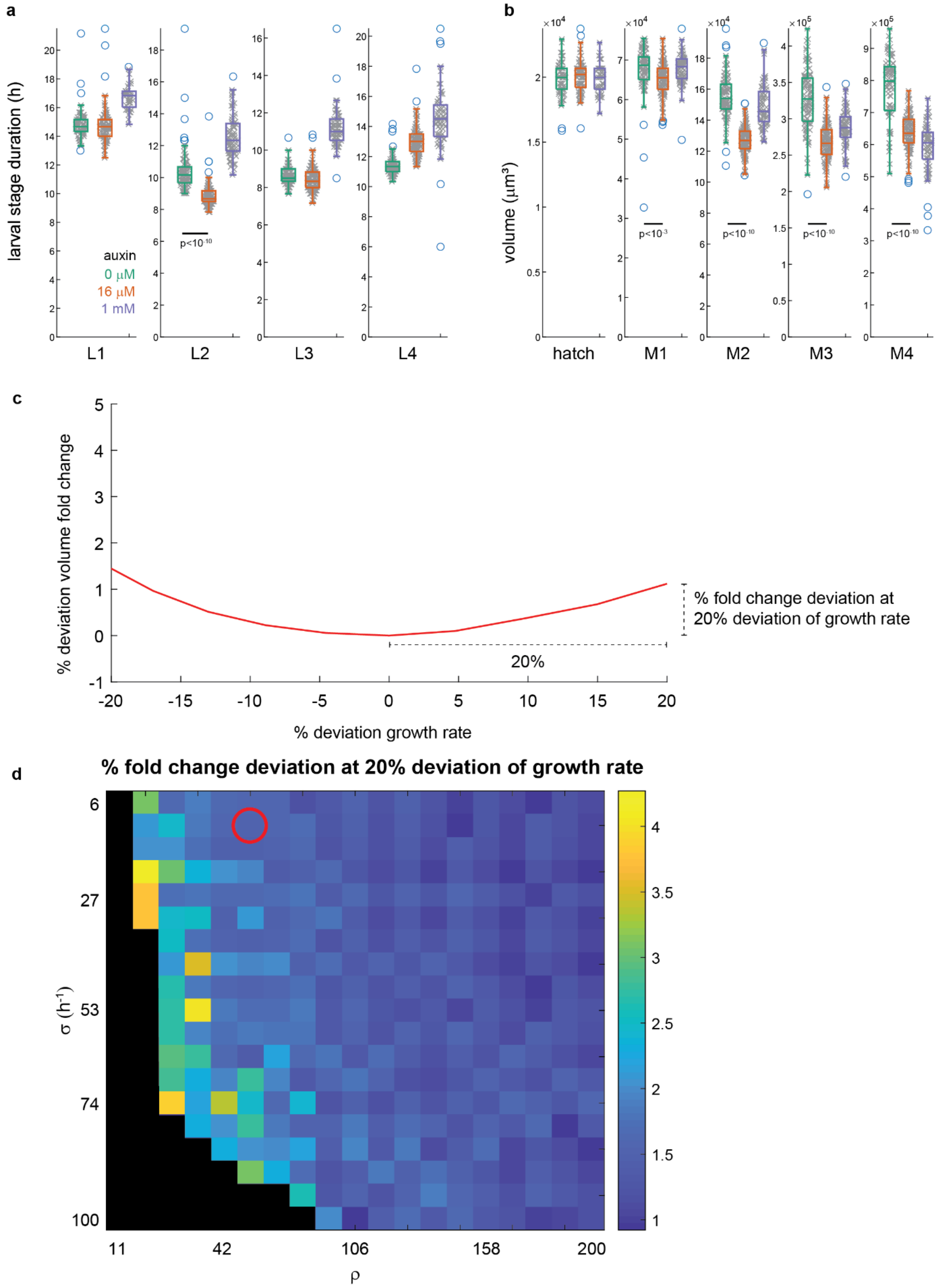

Supplementary Figure 10. Model behaviour is robust to changes of parameter values

(related to Figure 7)

a. Box plot of the larval stage duration of a *blmp-1::aid*, *eft-3p::tir1* strain at indicated auxin concentration. The larval stage duration is significantly reduced at L2 stage when animals are exposed to low levels of auxin, consistent with a higher frequency of *dpy-9p::gfp* oscillations (see Fig. 7). A weak effect is also observed at L3 stage. High dose of auxin delays development due to loss of function of BLMP-1. The growth delay observed at low dose of auxin during L4 may indicate a larval stage specific function of BLMP-1, or be due to technical reasons, e.g. a dependence of auxin induced acceleration of protein degradation on the larval stage. p-values is one-sided t-tests (exact value: 1e-22). Crosses are measurements for individual animals.

b. Same as (a), but for volumes at hatch and larval moults. Low and high dose of auxin causes a systematically smaller size at all larval moults. p-values of one-sided t-tests are indicated for the comparison between 0mM auxin and 0.016 mM (exact p-values: 2e-4, 4e-42, 5e-27, 1e-26).

c. Illustration of the deviation in fold change plotted in (d). The volume fold change in the model was computed for a range of parameter values for ρ and σ as a function of μ. For each pair of ρ and σ the growth rate μ_0 was determined, for which the fold change, and hence the first derivative of the fold change with respect to μ was minimized (μ_0 is the μ, at which $\frac{\mathrm{d\,FCv}(\mu)}{\mathrm{d}\mu} = 0$). Subsequently, the relative change in fold change after increasing or decreasing μ by 20% was computed. (d) shows the larger one of the two values.

d. Volume fold change deviation from reference at a 20% change of μ for a range of model parameters. Deviation is less than 2% from reference for a large parameter space. Black areas are parameter values for which the system does not oscillate. Red circle indicates parameters used in Figures 7c-f.

a, b. Boxplots: central line: median, box: interquartile ranges (IQR), whisker: ranges except extreme outliers (>1.5*IQR). crosses: individual observations. Number of animals (n) for L1 to L4 from number of independent experiments (m): 0mM IAA: n = 71, 92, 92, 83; m = 3. 16µM IAA: n = 93, 120, 118, 109; m = 3. 1mM IAA: n = 43, 73, 71, 68; m = 3.

Supplementary Table 1.

**volume**	hatch	M1	M2	M3	M4
eat-2	8.11e-01	6.99e-03	6.99e-03	6.99e-03	2.80e-02
dbl-1++	7.58e-01	3.03e-02	3.03e-02	1.82e-01	1.21e-01
lon-1	4.11e-04	4.63e-03	4.17e-01	8.87e-01	7.40e-01
raga-1	1.12e-01	7.69e-02	2.17e-01	6.99e-03	6.99e-03
lin-14	3.03e-02	3.03e-02	3.03e-02	3.03e-02	n/a

**length**	hatch	M1	M2	M3	M4
eat-2	1.40e-02	5.73e-01	6.99e-03	1.40e-02	6.99e-03
dbl-1++	3.03e-02	3.03e-02	3.03e-02	3.03e-02	3.03e-02
lon-1	8.87e-01	1.03e-04	1.03e-04	1.03e-04	1.03e-04
raga-1	4.69e-01	7.69e-02	2.87e-01	1.12e-01	6.99e-03
lin-14	6.06e-02	3.03e-02	1.21e-01	3.03e-02	n/a

**CV growth rate**	L1	L2	L3	L4
eat-2	6.99e-03	6.99e-03	6.99e-03	4.90e-02
dbl-1++	6.06e-02	1.21e-01	1.82e-01	3.64e-01
lon-1	1.93e-01	9.67e-03	4.75e-01	3.64e-01
raga-1	6.99e-03	6.99e-03	2.80e-02	7.69e-02
lin-14	3.03e-02	4.85e-01	1.21e-01	n/a

p-values of comparisons between mutants and wild type as shown in Figures 5b-d.

Supplementary Table 2

	**L1**	**L2**	**L3**	**L4**
**wild type**	-0.43+/-0.02	-0.69+/-0.02	-0.39+/-0.02	-0.26+/-0.01
eat-2	-0.45+/-0.03	-0.63+/-0.03	-0.44+/-0.03	-0.25+/-0.02
**DBL-1 +++**	-0.49+/-0.02	-0.98+/-0.02	-0.26+/-0.02	-0.25+/-0.01
lon-1	-0.5+/-0.03	-0.99+/-0.03	-0.41+/-0.02	-0.19+/-0.02
raga-1	-0.64+/-0.03	-1.05+/-0.03	-0.54+/-0.02	-0.4+/-0.02
lin-14	-0.78+/-0.05	-0.54+/-0.05	-0.44+/-0.03	n/a

Slope +/- SEM of the relation between the growth rate and the larval stage duration shown in Figure 5e and Supplementary Figure 8.

Supplementary Table 3

	**L1**	**L2**	**L3**	**L4**
**wild type**	1.52e-81	4.4e-192	3.8e-113	4.3e-138
eat-2	1.78e-34	3.95e-63	5.79e-45	1.08e-41
**DBL-1 +++**	6.3e-70	1.2e-190	2.86e-35	4.45e-60
lon-1	8.03e-52	2.7e-155	2.9e-66	7.41e-25
raga-1	4.01e-72	2.3e-147	1.99e-74	6.46e-71
lin-14	7.29e-36	1.31e-24	5.12e-37	n.a.

p-value for negative correlation between the growth rate and the larval stage duration shown in Figure 5e and Supplementary Figure 8.
