# Supplementary material for: Coupling of growth rate and developmental tempo reduces body size heterogeneity in C. elegans
Source: Nat Commun. 2022 Jun 6;13:3132. doi: 10.1038/s41467-022-29720-8 (PMC9170734; doi:10.1038/s41467-022-29720-8)
Supplement: Supplementary file 3 — Reporting Summary [file 41467_2022_29720_MOESM3_ESM.pdf]

nature portfolio

Corresponding author(s): Benjamin D. Towbin, Helge Grosshans

Last updated by author(s): Mar 1, 2022

Reporting Summary

Nature Portfolio wishes to improve the reproducibility of the work that we publish. This form provides structure for consistency and transparency in reporting. For further information on Nature Portfolio policies, see our Editorial Policies and the Editorial Policy Checklist.

Statistics

For all statistical analyses, confirm that the following items are present in the figure legend, table legend, main text, or Methods section.

n/a	Confirmed	
☐	☒	The exact sample size (*n*) for each experimental group/condition, given as a discrete number and unit of measurement
☐	☒	A statement on whether measurements were taken from distinct samples or whether the same sample was measured repeatedly
☐	☒	The statistical test(s) used AND whether they are one- or two-sided<br>*Only common tests should be described solely by name; describe more complex techniques in the Methods section.*
☒	☐	A description of all covariates tested
☐	☒	A description of any assumptions or corrections, such as tests of normality and adjustment for multiple comparisons
☐	☒	A full description of the statistical parameters including central tendency (e.g. means) or other basic estimates (e.g. regression coefficient) AND variation (e.g. standard deviation) or associated estimates of uncertainty (e.g. confidence intervals)
☐	☒	For null hypothesis testing, the test statistic (e.g. *F*, *t*, *r*) with confidence intervals, effect sizes, degrees of freedom and *P* value noted<br>*Give P values as exact values whenever suitable.*
☒	☐	For Bayesian analysis, information on the choice of priors and Markov chain Monte Carlo settings
☒	☐	For hierarchical and complex designs, identification of the appropriate level for tests and full reporting of outcomes
☐	☒	Estimates of effect sizes (e.g. Cohen's *d*, Pearson's *r*), indicating how they were calculated

Our web collection on statistics for biologists contains articles on many of the points above.

Software and code

Policy information about availability of computer code

Data collection	No software was used for data collection.
Data analysis	Data analysis was conducted using custom made Matlab scripts (v2021b, https://github.com/btowbin/NatComm2022) and an FMI-specific Fiji plugin (fmi-ij2-plugins, https://zenodo.org/record/3560533#.Yhf2GejML8A) in combination with KNIME (v3.7.2).

For manuscripts utilizing custom algorithms or software that are central to the research but not yet described in published literature, software must be made available to editors and reviewers. We strongly encourage code deposition in a community repository (e.g. GitHub). See the Nature Portfolio guidelines for submitting code & software for further information.

Data

Policy information about availability of data

All manuscripts must include a data availability statement. This statement should provide the following information, where applicable:

- Accession codes, unique identifiers, or web links for publicly available datasets
- A description of any restrictions on data availability
- For clinical datasets or third party data, please ensure that the statement adheres to our policy

The size, growth, and fluorescence data generated in this study are provided in the Supplementary Information and Source Data file.

Field-specific reporting

Please select the one below that is the best fit for your research. If you are not sure, read the appropriate sections before making your selection.

☒ Life sciences ☐ Behavioural & social sciences ☐ Ecological, evolutionary & environmental sciences

For a reference copy of the document with all sections, see nature.com/documents/nr-reporting-summary-flat.pdf

Life sciences study design

All studies must disclose on these points even when the disclosure is negative.

Sample size	No sample size calculations were performed. The sample sizes in the study were chosen based on prior experience and reasonable replication. Number of animals and independent repeats are indicated in the figure legends.
Data exclusions	No data was excluded.
Replication	All experiments were replicated independently on multiple days (at least 2, up to 10).
Randomization	Samples were allocated into experimental groups based on genotype and/or supplement. Samples were grown in parallel in the presence and absence of treatment.
Blinding	Blinding was not possible during data collection because the acquired experimental data required manipulations of live animals. However, data analysis was fully automated.

Reporting for specific materials, systems and methods

We require information from authors about some types of materials, experimental systems and methods used in many studies. Here, indicate whether each material, system or method listed is relevant to your study. If you are not sure if a list item applies to your research, read the appropriate section before selecting a response.

Materials & experimental systems

n/a	Involved in the study	
☒	☐	Antibodies
☒	☐	Eukaryotic cell lines
☒	☐	Palaeontology and archaeology
☐	☒	Animals and other organisms
☒	☐	Human research participants
☒	☐	Clinical data
☒	☐	Dual use research of concern

Methods

n/a	Involved in the study	
☒	☐	ChIP-seq
☒	☐	Flow cytometry
☒	☐	MRI-based neuroimaging

Animals and other organisms

Policy information about studies involving animals; ARRIVE guidelines recommended for reporting animal research

Laboratory animals	The C. elegans laboratory strain (N2 Bristol) and deletion mutants thereof as detailed in the Methods section wwer used in this study. C. elegans are predominantly self-fertilizing hermaphrodites grown from birth to day 3 for experimentation in this publication.
Wild animals	no wild animals were involved
Field-collected samples	the study did not involve field-collected samples
Ethics oversight	no ethical approval was required as the study was concerned with C. elegans exclusivels

Note that full information on the approval of the study protocol must also be provided in the manuscript.
